# Cesarean Section and Socioeconomic Status in Tehran, Iran 

**Published:** 2017-09-12

**Authors:** Reza Omani-Samani, Maryam Mohammadi, Amir Almasi-Hashiani, Saman Maroufizadeh

**Affiliations:** ^1^ Department of Epidemiology and Reproductive Health, Reproductive Epidemiology Research Center, Royan Institute for Reproductive Biomedicine, ACECR, Tehran, Iran

**Keywords:** Cesarean section, Prevalence, Socioeconomic status, Iran

## Abstract

**Background:** Socioeconomic status (SES) is linked to a wide range of maternity services including Cesarean section (CS). The objective of this study was to determine the rate of CS and to examine the effect of SES on CS rate.

**Study design:** Cross-sectional study.

**Methods:** This study included 4308 pregnant women who gave singleton birth in Tehran, Iran in July 2015. To evaluate the effect of SES on CS, logistic regression model was used after adjusting for others variables.

**Results:** The CS rate was 72.0% and its rate in private hospitals was significantly higher than in public hospitals (91.7 vs 62.6%, *P*<0.001). After adjusting for demographic characteristics of mothers, obstetrical data and newborn’s information, economic status (ES) was associated with an increased rate of CS (OR= 1.22; 95% CI=1.16–1.28).

**Conclusions:** Independently of biological or clinical factors, ES is associated with an increased rate of CS in Tehran, Iran.

## Introduction

Over the last few decades, Cesarean section (CS) rate has increased significantly worldwide, especially in Iran. Based on the latest data from 150 countries, the CS rate was 18.6% (1). The highest and the lowest rate of CS were reported in Latin America and the Caribbean (40.5%) and Africa (7.3%)^1^.

The CS has some complications for the mother and infant. After CS, the most common complication includes bleeding, infection, postpartum hemorrhage, wound infection and endometritis ^2,3^. CS risks for the infant include breathing problems, low Apgar scores, and fetal injury^4^. Socioeconomic status (SES), whether assessed by income, education, or occupation, is an important determinant of health outcomes. It influences utilization of various available health facilities such as CS. In two studies conducted in China and Brazil, the SES was related to CS ^5, 6^. The objective of this study was to determine the rate of CS among pregnant women and to examine the effect of SES on CS rate.

## Methods

This cross sectional study was conducted on 4308 pregnant women with singleton live births referred to maternity hospitals (46 public and 30 private hospitals) in Tehran, Iran in July 2015. The eligibility criteria for this study were as follows: (a) life birth, and (b) singleton pregnancy. A checklist was provided to collect the data on demographic characteristics of mothers, obstetrical data, and newborn’s information. This information includes mother’s age, mother’s education, father education, mother’s occupation, ES, body mass index, parity, history of abortion, history of still birth, pre-eclampsia, use of assisted reproductive technology, infant sex, infant weight, infant height, baby’s head circumference and type of hospital.

Furthermore, a principal component analysis was conducted on questionnaires pertained to home appliances, digital goods, and to measure the economic status (ES) of each family.

The Ethics Committee of Royan Institute, Tehran, Iran, approved the protocol study (Code: IR. ACECR. Royan.REC.1395.43). The aim of the study was explained to the mothers and a written informed consent was obtained before data collection.

All the analyses were performed with IBM SPSS Statistics for Windows (IBM Crop., Armonk, NY, USA, ver. 22.0). Continuous variables were expressed as mean ± standard deviation (SD) and categorical variables as number (percentage). To evaluate the effect of SES on CS, logistic regression model was used after adjusting for others variables. All statistical tests were two-sided and level of significance was set at 0.05.

## Results

Overall, 4308 deliveries (public hospitals: n=2921, 67.8% and private hospitals: n=1387, 32.2%) of live-born singletons were investigated. The mean age of mothers was 29.14 ±5.33 yr and the mean of parity was 1.65 ±0.75. Among all women, 32.6% had university education, 19.3% had history of abortion, 1.8% had history of stillbirth, 5.1% had preeclampsia, 12.0% were employed, and 7.3% conceived with ART treatment. Of 4308 infants, 50.9% were male and mean birth weight and baby’s head circumference were 3215.3 ±443.3 gr and 34.89 ±4.88 cm, respectively.

The CS rate was 72.0%. The CS rates in private hospitals was significantly higher than in public hospitals (91.7 vs 62.6%, *P*<0.001) (Table 1). In univariate analysis, mother’s education (odds ratio (OR) =2.74; 95% confidence interval (95% CI): 2.33, 3.22), father’s education (OR=2.68; 95% CI: 2.27, 3.15), mother’s occupation (OR=3.19; 95% CI: 2.42, 4.19) and ES (OR=1.42; 95% CI: 1.37, 1.48) were associated with CS. Moreover, after adjusting for demographic characteristics of mothers, obstetrical data and newborn’s information, only ES was associated with an increased rate of CS (OR=1.22; 95% CI: 1.16, 1.28) (Table 1).

**Table 1:** Association between Cesarean section and some socioeconomic factors among pregnant women in Tehran, Iran

**Variables**	**Cesarean section**	**Vaginal delivery**	**Univariate analysis**		**Multivariate analysis**	
			**Crude OR (95% CI)**	***P*** **value**	**Adjusted OR (95% CI)**	***P*** **value**
Type of hospital						
Public (%)	1828 (62.6)	1093 (37.4)	1.00		1.00	
Private (%)	1272 (91.7)	115 (8.3)	6.61 (5.39, 8.12)	0.001	4.11 (3.30, 5.11)	0.001
Mother's education						
Non-Academic (%)	1917 (66.0)	986 (34.0)	1.00		1.00	
Academic (%)	1183 (84.2)	222 (15.8)	2.74 (2.33, 3.22)	0.001	1.13 (0.91, 1.40)	0.268
Father's education						
Non-Academic (%)	1946 (66.3)	989 (33.7)	1.00		1.00	
Academic (%)	1154 (84.0)	219 (16.0)	2.68 (2.27, 3.15)	0.001	1.09 (0.88, 1.35)	0.423
Mother's occupation						
Housewife (%)	2644 (69.8)	1146 (30.2)	1.00		1.00	
Employed (%)	456 (88.0)	62 (12.0)	3.19 (2.42, 4.20)	0.001	1.11 (0.81, 1.53)	0.515
Economic status (SD)	0.37 (2.06)	-0.88 (1.63 )	1.42 (1.37, 1.48)	0.001	1.22 (1.16, 1.28)	0.001

## Discussion

In the present study, the rate of CS was 72.0%, which is substantially higher than the worldwide average, which is 18.6%^1^. These differences may be due to different cultures, incorrect attitudes toward vaginal delivery, fashions in Iranian women, lack of facilities for vaginal delivery and behavior of health care personnel.

Consistent with prior literature, the CS rates in private hospitals was substantially higher than in public hospital. In Brazil, the rate of CS was 4.4 times higher at a private hospital than at a public one^7^. Based on adjusted analysis, ES was a strong predictor for CS, which in line with previous studies^5, 6^. In other studies, mother’s occupation, high education, and high income were the most important nonclinical variables that affected CS^8, 9^.

The present study had several limitations. First, because of the cross-sectional nature of the study, causal inferences cannot be established. Second, our study was conducted only in Tehran; therefore, the results cannot be generalized to other cities in Iran. Despite these limitations, this study is a first known study to examine the effect of SES on CS rate in capital city of Iran using a large representative data.

## Conclusions

The prevalence of CS was substantially high, especially in private hospitals, and dissimilar to other countries. Independently of biological or clinical factors, ES is associated with an increased rate of CS among pregnant women in Tehran, Iran.

## Acknowledgements

The authors would like to express their appreciation to those who participated in this study and to the staff at the hospitals of Tehran University of Medical Sciences, Shahid Beheshti University of Medical Sciences, Iran University of Medical Sciences and Islamic Azad University.

## Conflict of interest statement

The authors declared no conflicts of interest.

## Funding

This study was funded by Reproductive Epidemiology Research Center, Royan Institute for Reproductive Biomedicine, ACECR, Tehran, Iran.

## Highlights

- Economic status is associated with an increased rate of Cesarean section in Tehran, Iran.
- The Cesarean section rate in Tehran, Iran was substantially higher than the worldwide average.
- The Cesarean section rates in private hospitals were higher than in public hospitals.
